# The link between knowledge of the maternal diet and breastfeeding practices in mothers and health workers in Poland

**DOI:** 10.1186/s13006-021-00406-z

**Published:** 2021-08-09

**Authors:** Karolina Karcz, Izabela Lehman, Barbara Królak-Olejnik

**Affiliations:** Department and Clinic of Neonatology, Wroclaw Medical University, Wroclaw, Poland

**Keywords:** Breastfeeding, Lactation, Diet, Healthcare surveys

## Abstract

**Background:**

There are multiple misconceptions concerning the breastfeeding mother’s diet and its adverse impact on breast milk composition and the breastfed child’s health, which might lead to breastfeeding cessation. Although prophylactic maternal dietary restrictions are not recommended, mothers all over the world are often recommended to avoid certain foods, due to cultural beliefs, social pressure and even outdated or ambiguous medical recommendations. In Poland, there is no systematic approach to breastfeeding education in the form of nationwide educational programs for particular social groups. It was estimated that in 2017 only 3–4% of Polish infants were exclusively breastfed at 6 months of age. The aim of this study was to recognize the scale of common dietary misconceptions among lactating mothers in Poland and to compare knowledge and opinions between medical staff and mothers who have ever breastfed a child. In addition, the paper is an attempt to identify factors contributing to the still current practice of recommending prophylactic dietary restrictions to breastfeeding mothers by medical staff.

**Methods:**

The study was conducted in Poland, in January – February 2019. The study used a diagnostic poll method and was conducted mainly in an electronic form. A total of 1159 completed questionnaires data were analyzed: 35.1% completed by medical staff and 64.9% by mothers in non-medical professions. Statistical calculations were conducted with Chi-square test, logistic regression and U Mann Whitney test (level of significance set at 0.05).

**Results:**

The respondents presented a good level of knowledge and predominantly assessed the questioned statements correctly. Duration of breastfeeding was found to be the main factor determining respondents’ knowledge (*p* <  0.05). Concerning medical staff, the parity (*p* <  0.001) and applying an elimination diet when themselves breastfeeding (*p* <  0.001) had a significant impact on recommendation of prophylactic dietetic restrictions to the lactating women.

**Conclusions:**

Regardless of a resonably good level of knowledge on maternal nutrition in the lactation period, both breastfeeding mothers and medical staff are still convinced of the beneficial effect of preventive dietary restrictions, which affects further lactational counselling and lactational performance.

## Background

From the early stages of life, appropriate nutrition is essential to provide good physical and mental development and long-term health. Since mother’s milk composition is usually adjusted to the infant’s needs and is sufficient to ensure sustainable growth and development, breastfeeding is a standard nutrition for all infants (ESPGHAN) [1].

To compensate for both infant’s and mother’s demands, the process of lactation requires a greater need for nutrients. Maternal nutritional status and dietary intake mainly influence milk concentrations of vitamins (A, D, B12, thiamin, riboflavin, pyridoxine), iodine, selenium and fatty acids profiles, whereas the milk content of proteins, carbohydrates and other minerals remains unaltered, unless the mother is extremely undernourished or depleted in body stores (WHO, IOM) [2–4].

Exclusive breastfeeding, is recommended for 6 months, and when practiced for at least 3 to 4 months, is proven to reduce the incidence of atopic disease in infants (AAP) [5]. On the contrary, the concentrations of allergens in breast milk might be high enough to mediate the process of sensitization to antigens, therefore resulting in development of symptoms such as fussiness, hives, eczema, regurgitations, stool changes and other gastrointestinal disorders. However, the presence of these symptoms can be attributed to other reasons, not associated with hypersensitivity to food allergens (AAP) [5, 6]. Unfortunately, all these infantile ailments are sometimes considered to result from breastfeeding and the impact of maternal nutrition on breast milk quality [7]. Plenty of misconceptions have been present in societies and are often disseminated to consecutive generations, despite the changing state of scientific knowledge. As the issue of lactating women’s nutrition is overgrown with myths, mothers might receive contrary and ambiguous recommendations, as well as being urged to avoid certain foods because of cultural beliefs [8–11]. In many settings, mothers are constantly being informed that their breast milk can have adverse effects on infants, unless they change their dietary habits and follow a prophylactic elimination diet during breastfeeding. However, this practice might have a negative impact on breastfeeding rates.

In Poland, there is no systematic approach to breastfeeding education in the form of nationwide educational programs for particular social groups. Lactational education for medical staff is organized mainly as postgraduate nonobligatory courses. In 1995, breastfeeding was included for the first time in the National Health Program. The National Health Program in Poland is implemented and modified every 5 years and its strategic goal is to improve the health of the population, including the promotion of proper nutrition for children. According to the National Health Program for 2016–2020 [12], all efforts should have been made to improve health care for the mother and infant, including improving nutritional status and breastfeeding rates. In this matter, the overarching aim was to take all appropriate measures to ensure the success of breastfeeding, including the identification of risk factors for early cessation of breastfeeding and the early identification of potential breastfeeding problems. The introduction of the Standard of Perinatal Care in 2012 [13] was supposed to be a breakthrough in the promotion of breastfeeding in Poland. Now all maternity hospitals are obliged to protect, promote and support breastfeeding. However, a nationwide study reported that at 2 months of age, only 43% of infants were still breastfed, and at 6 months of age, only 3–4% of infants were exclusively breastfed [14].

The available literature provides several studies conducted in different world regions, concerning the topic of myths attached to a mother’s diet during lactation [7–11]. However, none of them compares the level of knowledge and opinions on nutrition in the lactation period between the groups of patients and healthcare providers. This paper presents an overview of both Polish medical personnel and Polish breastfeeding mothers’ opinions regarding common misconceptions concerning lactation and nutrition. In addition, the impact of maternal experience with breastfeeding and dietary restrictions on both level of knowledge and further continuation of breastfeeding, as well as on applying personal experience in medical practice was indicated.

The aim of this study was to recognize the scale of common lactating mothers’ dietary misconceptions, mainly concerning the composition of human milk and its relationship to mother’s nutrition, adverse symptoms in infants, and popular misbeliefs regarding the consumption of particular products. A further objective of the survey was to assess and compare knowledge of respondents practicing medical and non-medical professions. In this context, the paper is an attempt to identify factors contributing to the still current practice of recommending prophylactic dietary restrictions to breastfeeding mothers by medical staff.

## Methods

### Study design

The study used the diagnostic poll method, and thus was based on a convenience sample, using a survey questionnaire previously compiled by the authors.

The study was designed only in the Polish language and conducted in Poland.

Prior to the start of the study, the comprehension of the questionnaire was tested during face-to-face interviews with 10 randomly selected multiparous mothers who were hospitalized with their infants after delivery in the authors’ clinic.

The questionnaire consisted of 3 parts: 1) questions regarding whether a breastfeeding mother can include particular products in her daily diet; 2) questions concerning popular myths regarding nutrition when breastfeeding and referring to the knowledge on breast milk composition; 3) demographic data and respondents’ experience with dietary restrictions. The questionnaire included only close-ended questions. Parts 1) and 2) consisted of single-choice questions (‘Yes / No’ or ‘True / False’). Part 3) included single-choice questions provided with a list of options. Answers to all questions were required.

In this paper, the results on the popularity of common myths, referring to the impact of mother’s nutrition on the process of lactation and infant’s health condition, are presented.

### Myths and beliefs

Based on worldwide publications, the most popular convictions on breastfeeding and nutrition in the lactation period concern the following aspects: human milk composition, impact of maternal diet on breast milk composition, impact of consumption of particular products on the quantity of milk and progress of lactation, adverse effect of mother’s diet on the infant’s condition, safety of consumption of particular meals by mother and her breastfed child [7–11]. For the purpose of this study, 23 statements were included in the survey and for the analysis, they were categorized in 3 groups, as follows: 1) impact of mother’s diet on breast milk composition; 2) general statements regarding mother’s diet during lactation; 3) mother’s diet - impact on the lactation process and infant’s well-being.

### Target group

The following groups were invited to participate in the study: 1) mothers pursuing professions outside the healthcare sector, who had any experience of breastfeeding, provided the child was delivered at term, regardless of the duration of lactation, including expressed breast milk feeding, representing society as a whole; 2) medical staff, regardless of age, sex and parity. The study did not differentiate exclusive breastfeeding from simultaneous feeding with both breast milk and infant formula.

### Data collection

In January–February 2019, the questionnaire was distributed in electronic form, mainly in social networks on randomly selected discussion forums and groups, identified via Facebook and other publicly available websites. These discussion forums and groups: 1) concerned the topics of breastfeeding and parenthood – those were visited by parents, mainly mothers, who were sharing their experiences in e.g. breastfeeding, weaning, child rearing; 2) were associated with medical staff (e.g. physicians, nurses, midwives, lactation consultants) – those were visited by medical staff who were sharing their experiences in e.g.: work with the patients, specialization training, additional postgraduate training or were sharing their knowledge of their fields of interest.

Advertisements including information on the study were published in each of the selected groups and forums. To ensure the eligibility of respondents: 1) all persons interested in participation in the study were contacted directly by a private message and after a short interview (concerning the performed profession, parity and experience in breastfeeding) provided with a link to the questionnaire; 2) the questionnaire included questions about: sex of the respondent, profession, parity, duration of breastfeeding of each of the offspring. Based on the completed questionnaire, if the eligibility of the respondent was questionable or objectionable, the answers were excluded from the final analysis.

All the participants were enrolled voluntarily, did not receive any remuneration and fully agreed to complete the questionnaire. Starting and completing the survey confirmed the respondents’ consent to participate in the study. The responses were coded and saved anonymously.

### Data analysis

Formal analysis of the final results included the Chi square test, logistic regression and U Mann Whitney test. For calculations Microsoft Excel for Office 365 (Microsoft, Redmond, WA, USA), Statistica 13.3 (StatSoft, Inc., Tulsa, OK, USA) and R version 3.6.2 (R Core Team, 2013. R Foundation for Statistical Computing, Vienna, Austria. URL http://www.R-project.org/.) were used. In analysis, the level of respondents’ knowledge was dependent on the percentage of correct answers.

### Ethics

Prior to the study commencement, formal permission was obtained from the Bioethics Committee at the Medical University in Wroclaw (No. KB 519/19).

## Results

### Characteristics of the respondents

A total of 1180 completed questionnaires were received. As 15 records indicated male respondents performing non-medical professions and six records identified nulliparous women employed outside the healthcare field, these respondents were considered as ineligible to participate in the study and data from these records was excluded from further analysis. The final sample included 1159 respondents, 752 of whom were not health professionals. Among the respondents working in the medical professions, 10 were men and 397 women, and with 81.1% mothers who have ever breastfed a child. More than a half of all respondents lived in cities with over 100,000 residents and were over 30 years old. Half of the participants had one child and more than 50% of all children were breastfed at least for 6 months. Detailed data on the respondents is provided in Table 1.

**Table 1:** Basic characteristics of respondents (*n* = 1159)

Variable	Number of respondents (%)	
	Medical (***n*** = 407; 35.12%)	Non-medical (***n*** = 752; 64.88%)
**Age in years**		
< 20	0	2 (0.27%)
20–29	183 (44.96%)	308 (40.96%)
30–39	165 (40.54%)	416 (55.32%)
≥ 40	59 (14.50%)	26 (3.46%)
**Sex**		
Female	397 (97.53%)	752 (100%)
Male	10 (2.47%)	n/a
**Place of residence**		
Village	77 (18.92%)	136 (18.09%)
Cities < 100,000 residents	105 (25.80%)	196 (26.06%)
Cities > 100,000 residents	225 (55.28%)	420 (55.85%)
**Parity**		
0	77 (18.92%)	n/a
1	167 (41.03%)	419 (55.72%)
2	139 (34.15%)	287 (38.16%)
3	24 (5.90%)	43 (5.72%)
4 or more	0	3 (0.40%)
**Duration of first child’s breastfeeding (months)**		
0–3	55 (13.51%)	117 (15.56%)
4–5	23 (5.65%)	71 (9.44%)
6–12	119 (29.24%)	208 (27.66%)
13–24	100 (24.57%)	263 (34.97%)
More than 24	25 (6.14%)	93 (12.37%)

*n/a* non applicable

### Impact of mother’s diet on breast milk composition

**Fig. 1:**
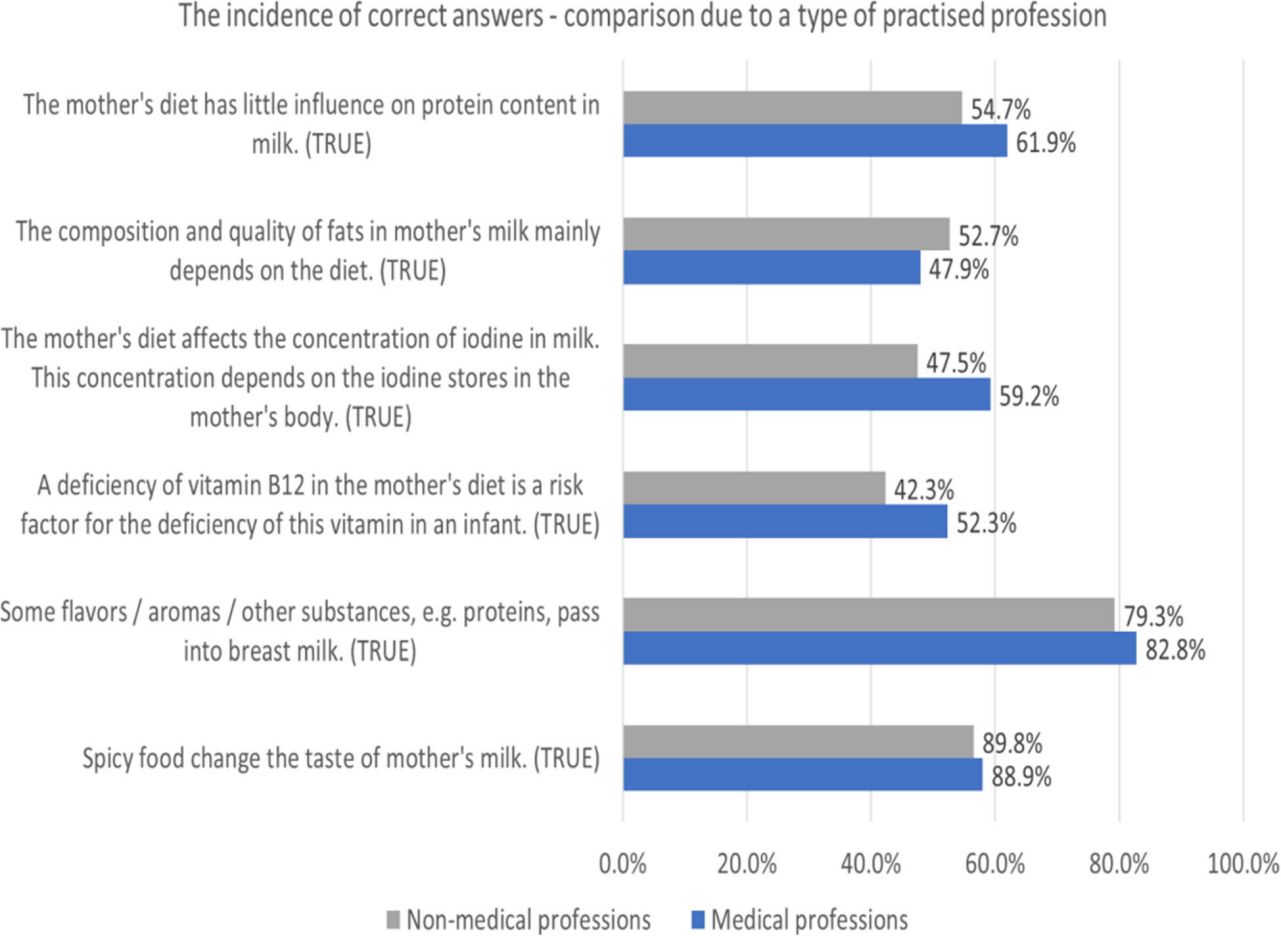
Impact of diet on breast milk composition. Incidence of correct answers - comparison due to type of profession

In general, respondents presented a quite low level of knowledge of human milk composition and its dependence on the mother’s nutrition. As far as the macronutrients are concerned, a total of 57.2% of all respondents answered correctly that dietary habits do not influence the concentration of breast milk proteins significantly, whereas 51% of all respondents had knowledge that the lipid profile of breast milk is influenced by the diet. In the case of micronutrients, only 51.6% of the study group knew the relationship between diet, maternal body stores and iodine concentration in human milk and barely 45.8% recognized an insufficient supply of vitamin B12 in mother’s diet as a risk factor for its deficiency in the infant. Even though respondents knew quite well that some substances, including flavors might pass to the mother’s milk (80.5%), only 57% indicated correctly that the taste of breastmilk can be affected by spicy food. The logistic regression analyses revealed several factors that were associated with correct answers among participants, with various directions of relationship, the most important being a medical professional, duration of breastfeeding and parity. These results are summarized in Table 2. Some differences in proportion providing correct answers were found between healthcare providers and breastfeeding mothers (Fig. 1).

**Table 2:** Factors influencing knowledge about the dietary impact on breast milk composition (*n* = 1159)

Variable	Univariate analysis		Multivariate analysis		Correct answer
	B; OR (95% CI)	***P***-value	B; OR (95% CI)	***P***-value	
**The mother’s diet has little influence on protein content in milk.**					TRUE
Parity	0.115; 1.12 (0.95, 1.32)	0.168	n/a	n/a	
Duration of breastfeeding the first child	0.017; 1.02 (1.0, 1.03)	0.012^a^	0.018; 1.02 (1.0, 1.03)	0.007^a^	
Applied an elimination diet during breastfeeding	−0.25; 0.78 (0.6, 1.01)	0.061	n/a	n/a	
Used elimination diet and replaced breastfeeding with infant formula	−0.344; 0.71 (0.41, 1.23)	0.222	n/a	n/a	
Being a medical professional	0.299; 1.35 (1.05, 1.73)	0.017^a^	0.421; 1.52 (1.16, 2.0)	0.002^a^	
Applied an elimination diet according to a doctor’s recommendations	−0.058; 0.94 (0.7, 1.27)	0.699	n/a	n/a	
**The composition and quality of fats in mother’s milk mainly depends on the diet.**					TRUE
Parity	−0.156; 0.86 (0.73, 1.01)	0.06	n/a	n/a	
Duration of breastfeeding the first child	−0.001; 0.999 (0.99, 1.01)	0.825	n/a	n/a	
Applied an elimination diet during breastfeeding	0.007; 1.01 (0.78, 1.31)	0.958	n/a	n/a	
Used elimination diet and replaced breastfeeding with infant formula	0.4; 1.49 (0.85, 2.62)	0.164	n/a	n/a	
Being a medical professional	−0.190; 0.83 (0.65, 1.05)	0.123	n/a	n/a	
Applied an elimination diet according to a doctor’s recommendations	−0.194; 0.82 (0.61, 1.1)	0.194	n/a	n/a	
**The mother’s diet affects the concentration of iodine in milk. This concentration depends on the iodine stores in the mother’s body.**					TRUE
Parity	−0.294; 0.75 (0.63, 0.88)	< 0.001^a^	−0.254; 0.78 (0.66, 0.92)	0.003^a^	
Duration of breastfeeding the first child	−0.007; 0.994 (0.98, 1.01)	0.304	n/a	n/a	
Applied an elimination diet during breastfeeding	−0.009; 0.991 (0.765, 1.29)	0.948	n/a	n/a	
Used elimination diet and replaced breastfeeding with infant formula	0.211; 1.24 (0.71, 2.15)	0.456	n/a	n/a	
Being a medical professional	0.474; 1.61 (1.26, 2.05)	< 0.001^a^	0.419; 1.52 (1.19, 1.95)	< 0.001^a^	
Applied an elimination diet according to a doctor’s recommendations	0.021; 1.02 (0.76, 1.37)	0.888	n/a	n/a	
**A deficiency of vitamin B12 in the mother’s diet is a risk factor for the deficiency of this vitamin in an infant.**					TRUE
Parity	−0.280; 0.76 (0.64, 0.89)	< 0.001^a^	− 0.276; 0.76 (0.62, 0.92)	0.006^a^	
Duration of breastfeeding the first child	−0.019; 0.98 (0.97, 0.99)	0.004^a^	−0.019; 0.98 (0.97, 0.99)	0.005^a^	
Applied an elimination diet during breastfeeding	0.099; 1.1 (0.85, 1.43)	0.457	n/a	n/a	
Used elimination diet and replaced breastfeeding with infant formula	−0.184; 0.83 (0.48, 1.46)	0.52	n/a	n/a	
Being a medical professional	0.404; 1.5 (1.18, 1.91)	0.001^a^	0.372; 1.45 (1.11, 1.89)	0.006^a^	
Applied an elimination diet according to a doctor’s recommendations	−0.26; 0.97 (0.73, 1.31)	0.861	n/a	n/a	
**Some flavors / aromas / other substances, e.g. proteins, pass into breast milk**.					TRUE
Parity	0.01; 1.01 (0.82, 1.24)	0.922	n/a	n/a	
Duration of breastfeeding the first child	0.020; 1.02 (1.0, 1.04)	0.022^a^	0.020; 1.02 (1.0, 1.04)	0.021^a^	
Applied an elimination diet during breastfeeding	0.386; 1.47 (1.04, 2.09)	0.03^a^	0.242; 1.38 (0.97, 1.96)	0.418	
Used elimination diet and replaced breastfeeding with infant formula	−0.309; 0.73 (0.39, 1.4)	0.346	n/a	n/a	
Being a medical professional	0.231; 1.26 (0.92, 1.72)	0.146	n/a	n/a	
Applied an elimination diet according to a doctor’s recommendations	0.403; 1.5 (1.0, 2.24)	0.05^a^	0.126; 1.42 (0.95, 2.14)	0.713	
**Spicy food changes the taste of mother’s milk.**					TRUE
Parity	−0.122; 0.89 (0.75, 1.04)	0.143	n/a	n/a	
Duration of breastfeeding the first child	0.005; 1.01 (0.99, 1.02)	0.427	n/a	n/a	
Applied an elimination diet during breastfeeding	0.677; 1.97 (1.5, 2.59)	< 0.001^a^	−1.08; 1.97 (1.5, 2.59)	< 0.001^a^	
Used elimination diet and replaced breastfeeding with infant formula	0.145; 1.16 (0.66, 2.03)	0.615	n/a	n/a	
Being a medical professional	0.06; 1.06 (0.83, 1.36)	0.63	n/a	n/a	
Applied an elimination diet according to a doctor’s recommendations	0.438; 1.55 (1.14, 2.1)	0.005^a^	0.55; 1.55 (1.14, 2.1)	0.055	

*B* Coefficient, *OR* Odds Ratio, *CI* Confidence Interval, *n/a* non applicable

^a^statistically significant

### General statements regarding mother’s diet during lactation

The vast majority of respondents (93.2%) considered that the diet during lactation should be well-balanced, with general recommendations as for every other adult. However, 73.7% of all participants knew that there are no specific dietary recommendations, apart from covering the higher needs for particular nutrients. Generally, the study group correctly supported the statement that only relevant medical indications are the basis for an elimination diet (94.2%) and that prophylactic dietary restrictions in a breastfeeding mother would not prevent their infants from developing food allergies or digestive tract disorders (92.4%). In general, respondents were aware that daily caloric and fluid demand increases during lactation (95.1%), but that the mother is not allowed to consume meals without any limits (92.9%). Nevertheless, there is no need to choose only light meals and to avoid fried nor hard to digest products (incidence of correct answers = 83.7%).

**Fig. 2:**
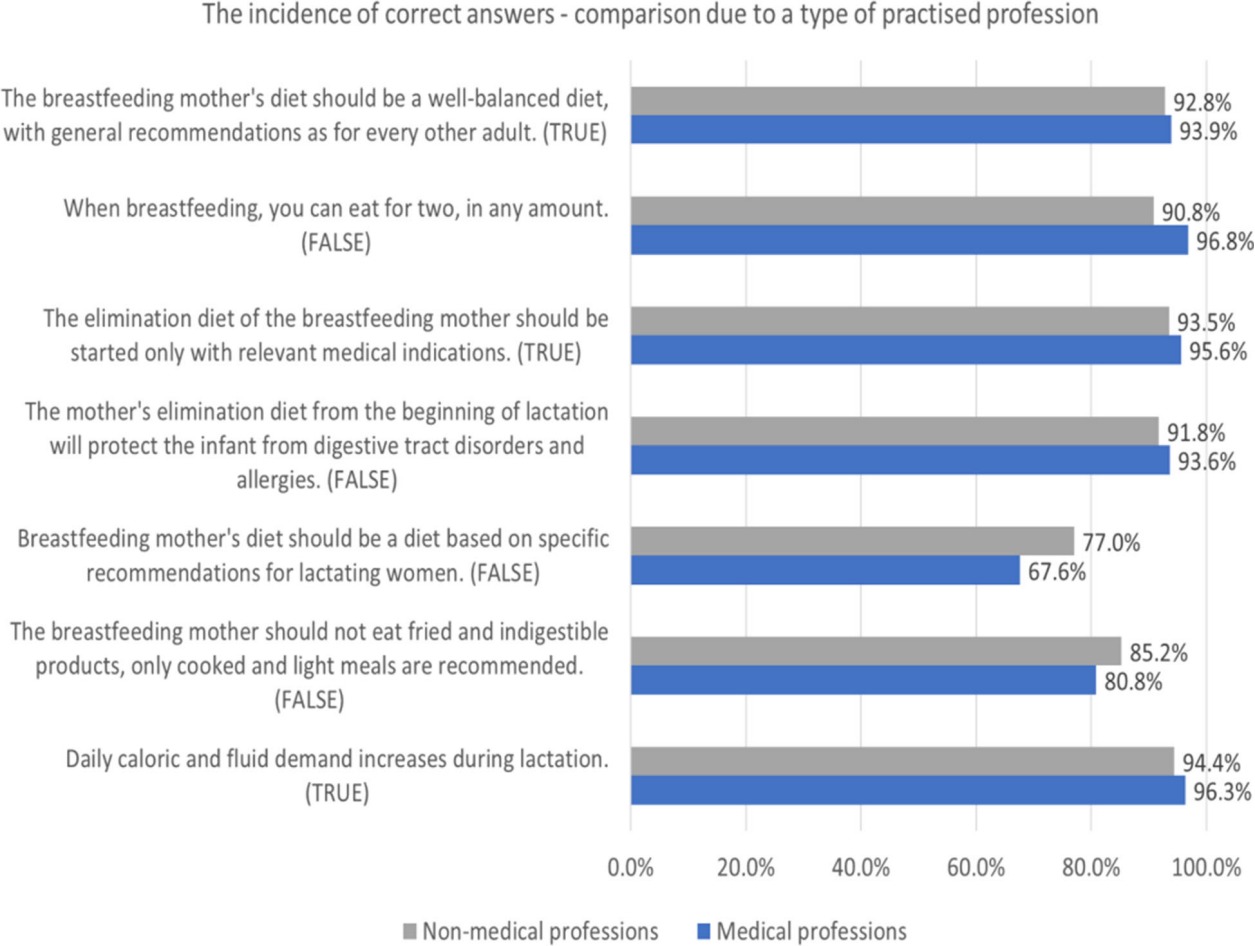
General statements regarding mother’s diet during lactation. Incidence of correct answers - comparison due to type of profession

The main factors affecting significantly (*p* <  0.05) participants’ opinions on whether the statements were true or false included: themselves having used an elimination diet during breastfeeding, applying an elimination diet due to their doctor’s recommendations and practicing a medical profession. The impact of these factors was found to vary across the questioned items, in these terms, the specific factor affected the prevalence of correct answers positively or negatively. The results have been summarized in Table 3. A comparison of the proportions of correct answers between healthcare providers and mothers is presented in Fig. 2.

**Table 3:** Factors influencing opinions on general statements regarding mother’s diet during lactation (*n* = )

Variable	Univariate analysis		Multivariate analysis		Correct answer
	B; OR (95% CI)	***P***-value	B; OR (95% CI)	***P***-value	
**The breastfeeding mother’s diet should be a well-balanced diet, with general recommendations as for every other adult.**					TRUE
Parity	−0.248; 0.78 (0.57, 1.07)	0.122	n/a	n/a	
Duration of breastfeeding the first child	0.009; 1.009 (0.98, 1.04)	0.524	n/a	n/a	
Applied an elimination diet during breastfeeding	−0.661; 0.52 (0.32, 0.83)	0.006^a^	n/a	n/a	
Used elimination diet and replaced breastfeeding with infant formula	−0.115; 0.89 (0.31, 2.54)	0.829	n/a	n/a	
Being a medical professional	0.167; 1.18 (0.72, 1.93)	0.504	n/a	n/a	
Applied an elimination diet according to a doctor’s recommendations	−0.231; 0.79 (0.46, 1.37)	0.409	n/a	n/a	
**When breastfeeding, you can eat for two, in any amount.**					FALSE
Parity	−0.258; 0.77 (0.57, 1.05)	0.102	n/a	n/a	
Duration of breastfeeding the first child	0.014; 1.01 (0.99, 1.04)	0.279	n/a	n/a	
Applied an elimination diet during breastfeeding	0.452; 1.57 (0.9, 2.76)	0.116	n/a	n/a	
Used elimination diet and replaced breastfeeding with infant formula	−0.073; 0.93 (0.33, 2.64)	0.891	n/a	n/a	
Being a medical professional	1.119; 3.06 (1.67, 5.61)	< 0.001^a^	1.149; 3.15 (1.72, 5.78)	< 0.001^a^	
Applied an elimination diet according to a doctor’s recommendations	0.699; 2.01 (0.99, 4.08)	0.053	0.763; 2.15 (1.05, 4.37)	0.035^a^	
**The elimination diet of the breastfeeding mother should be started only with relevant medical indications.**					TRUE
Parity	−0.079; 0.92 (0.66, 1.3)	0.652	n/a	n/a	
Duration of breastfeeding the first child	0.021; 1.02 (0.99, 1.05)	0.167	n/a	n/a	
Applied an elimination diet during breastfeeding	−0.576; 0.56 (0.34, 0.94)	0.027^a^	n/a	n/a	
Used elimination diet and replaced breastfeeding with infant formula	0.23; 1.02 (0.31, 3.37)	0.969	n/a	n/a	
Being a medical professional	0.41; 1.51 (0.87, 2.62)	0.147	n/a	n/a	
Applied an elimination diet according to a doctor’s recommendations	0.323; (0.69, 2.75)	0.358	n/a	n/a	
**The mother’s elimination diet from the beginning of lactation will protect the infant from digestive tract disorders and allergies.**					FALSE
Parity	−0.414; 0.66 (0.49, 0.89)	0.006^a^	−0.257; 0.66 (0.49, 0.89)	0.117	
Duration of breastfeeding the first child	0.013; 1.01 (0.99, 1.04)	0.285	n/a	n/a	
Applied an elimination diet during breastfeeding	−1.500; 0.22 (0.14, 0.35)	< 0.001^a^	−2.266; 0.11 (0.06, 0.2)	< 0.001^a^	
Used elimination diet and replaced breastfeeding with infant formula	−0.824; 0.44 (0.2, 0.96)	0.04^a^	−0.389; 0.44 (0.2, 0.96)	0.368	
Being a medical professional	0.275; 1.32 (0.82, 2.12)	0.256	n/a	n/a	
Applied an elimination diet according to a doctor’s recommendations	− 0.559; 0.57 (0.35, 0.93)	0.025^a^	1.222; 3.55 (1.89, 6.68)	< 0.001^a^	
**Breastfeeding mother’s diet should be based on specific recommendations for lactating women.**					FALSE
Parity	0.083; 1.09 (0.9, 1.31)	0.376	n/a	n/a	
Duration of breastfeeding the first child	0.138; 1.01 (1.0, 1.03)	0.066	n/a	n/a	
Applied an elimination diet during breastfeeding	−0.716; 0.49 (0.37, 0.65)	< 0.001^a^	−0.746; 0.47 (0.36, 0.63)	< 0.001^a^	
Used elimination diet and replaced breastfeeding with infant formula	0.099; 1.1 (0.58, 2.09)	0.762	n/a	n/a	
Being a medical professional	0.474; 0.62 (0.48, 0.81)	< 0.001^a^	−0.513; 0.6 (0.46, 0.79)	< 0.001^a^	
Applied an elimination diet according to a doctor’s recommendations	−0.231; 0.79 (0.58, 1.1)	0.16	n/a	n/a	
**The breastfeeding mother should not eat fried and indigestible products, only cooked and light meals are recommended.**					FALSE
Parity	−0.01; 0.991 (0.79, 1.23)	0.932	n/a	n/a	
Duration of breastfeeding the first child	0.045; 1.05 (1.02, 1.07)	< 0.001^a^	0.044; 1.04 (1.02, 1.07)	< 0.001^a^	
Applied an elimination diet during breastfeeding	−0.951; 0.39 (0.28, 0.53)	< 0.001^a^	1.461; 0.24 (0.14, 0.39)	< 0.001^a^	
Used elimination diet and replaced breastfeeding with infant formula	−0.312; 0.73 (0.37, 1.45)	0.371	n/a	n/a	
Being a medical professional	−0.314; 0.73 (0.53, 1.0)	0.053	n/a	n/a	
Applied an elimination diet according to a doctor’s recommendations	−0.505; 0.6 (0.42, 0.87)	0.006^a^	0.678; 1.94 (1.12, 3.35)	0.017^a^	
**Daily caloric and fluid demand increases during lactation.**					TRUE
Parity	−0.616; 0.54 (0.38, 0.77)	< 0.001^a^	n/a	n/a	
Duration of breastfeeding the first child	0.021; 1.02 (0.99, 1.05)	0.181	n/a	n/a	
Applied an elimination diet during breastfeeding	0.134; 1.14 (0.62, 2.12)	0.67	n/a	n/a	
Used elimination diet and replaced breastfeeding with infant formula	−0.747; 0.47 (0.18, 1.24)	0.128	n/a	n/a	
Being a medical professional	0.436; 1.55 (0.85, 2.82)	0.156	n/a	n/a	
Applied an elimination diet according to a doctor’s recommendations	0.555; 1.74 (0.78, 3.89)	0.177	n/a	n/a	

*B* Coefficient, *OR* Odds Ratio, *CI* Confidence Interval, *n/a* non applicable

^a^statistically significant

### Mother’s diet - impact on the lactation process and infant’s well-being

Almost all, up to 95.9% of respondents were found to believe that the crying of a breastfed infant results from insufficient breast milk supply, even if the lactation is stabilized, with sufficient milk production. As far as the popular beliefs regarding household methods of stimulating breast milk production are concerned, 88.5% of respondents correctly rebutted the effectiveness of drinking Bavarian tea (tea with milk). However, respectively only 39.9 and 29.2% of respondents contested the usefulness of drinking large amounts of water and beer. Mainly, the opinions were dependent on the duration of the lactation period and the prevalence of correct answers increased with the duration of breastfeeding.

Improper mother’s diet might be considered as another reason for crying of a breastfed infant. The great majority of respondents (96.3%) correctly considered this statement as false. Similarly, recognition of the belief that consuming spicy food or legumes by the mother might cause colic or bloating in a breastfed infant as untrue, was presented by 89.5 and 88.9% of respondents respectively. The logistic regression analysis revealed that the prevalence of correct answers was positively affected by the duration of the lactation period and negatively affected by applying an elimination diet while breastfeeding, regardless of reason for dietary restriction. About 95% of respondents correctly answered that drinking coffee during the lactation is allowed and it does not result in frequent infant waking at night.

Even though only 78.3% of respondents were aware that skin lesions located on a baby’s face do not always indicate an allergy to milk proteins, 93.4% of answers correctly stated that a breastfeeding mother is allowed to eat potentially allergenic products. In both cases, opinions were affected by the duration of lactation (positive impact on the prevalence of correct answers) and following an elimination diet in the period of lactation (negative impact on the prevalence of correct answers). In the case of the second statement, the presence or absence of medical advice was of key importance. The answers to the first of the aforementioned statements were also influenced by the type of profession, with higher rate of correct answers among medical professionals.

**Fig. 3:**
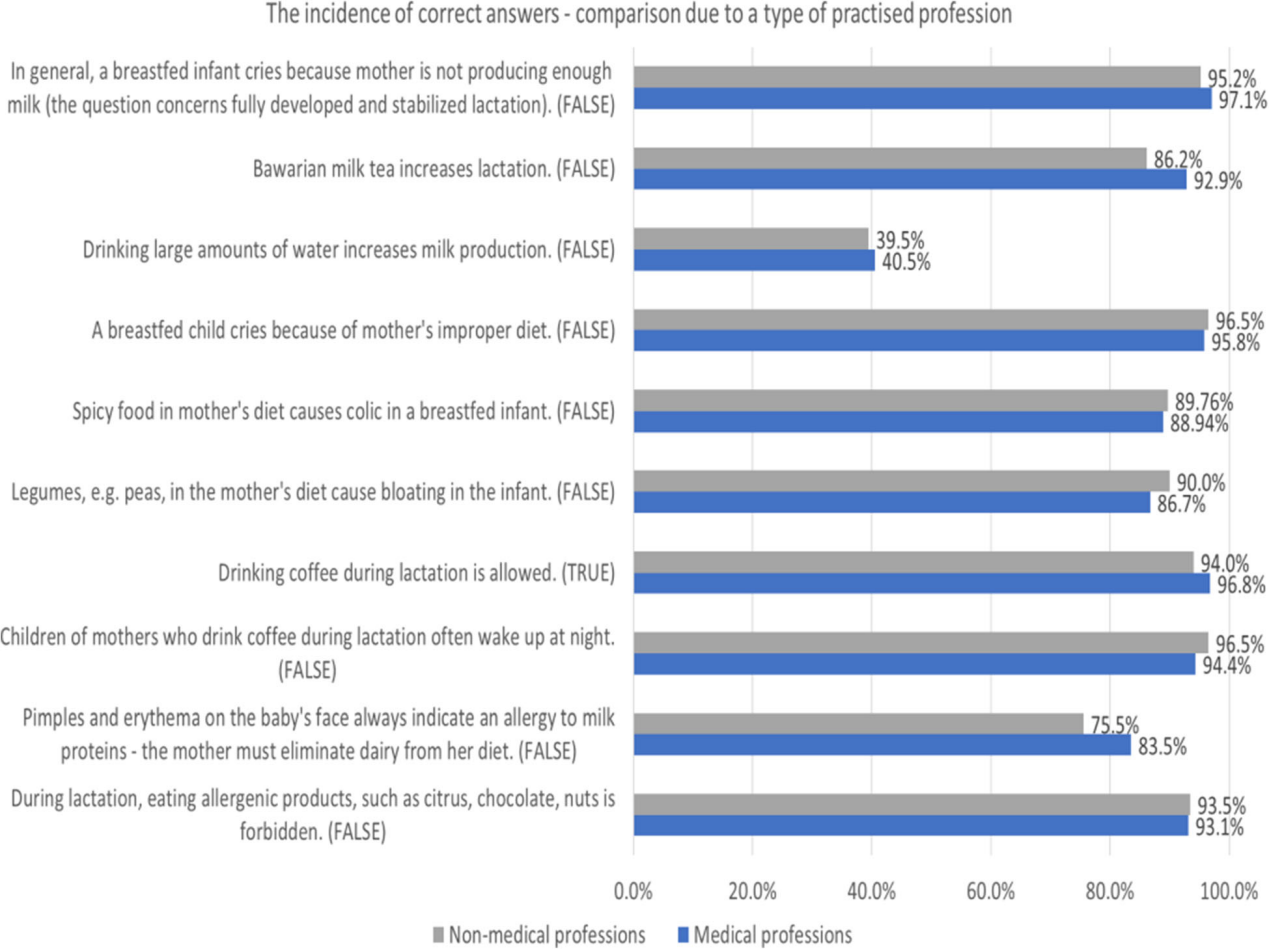
General statements regarding mother’s diet during lactation. Incidence of correct answers - comparison due to type of profession

The results of univariate and multivariate logistic regression analysis are summarized in Table 4. The comparison of prevalence of correct answers between healthcare providers and mothers has been presented in Fig. 3.

**Table 4:** Factors influencing knowledge regarding the impact of mother’s diet on the lactation process and infant’s well-being (*n* = 1159)

Variable	Univariate analysis		Multivariate analysis		Correct answer
	B; OR (95% CI)	***P***-value	B; OR (95% CI)	***P***-value	
**In general, a breastfed infant cries because is not getting enough mother’s milk (the question concerns fully developed and stabilized lactation).**					FALSE
Parity	0.059; 1.06 (0.71, 1.6)	0.776	n/a	n/a	
Duration of breastfeeding the first child	0.084; 1.09 (1.04, 1.14)	< 0.001^a^	0.084; 1.09 (1.04, 1.14)	< 0.001^a^	
Applied an elimination diet during breastfeeding	−0.507; 0.6 (0.33, 1.1)	0.098	n/a	n/a	
Used elimination diet and replaced breastfeeding with infant formula	− 1174; 0.31 (0.13, 0.76)	0.011^a^	− 0.823; 0.32 (0.13, 0.8)	0.081	
Being a medical professional	0.504; 1.66 (0.85, 3.22)	0.137	n/a	n/a	
Applied an elimination diet according to a doctor’s recommendations	0.333; 1.03 (0.49, 2.17)	0.93	n/a	n/a	
**Bavarian milk tea increases lactation.**					FALSE
Parity	−0.158; 0.85 (0.66, 1.1)	0.218	n/a	n/a	
Duration of breastfeeding the first child	0.027; 1.03 (1.01, 1.05)	0.015^a^	0.029; 1.03 (1.01, 1.05)	0.009^a^	
Applied an elimination diet during breastfeeding	−0.251; 0.78 (0.53, 1.15)	0.208	n/a	n/a	
Used elimination diet and replaced breastfeeding with infant formula	−0.333; 0.72 (0.33, 1.56)	0.399	n/a	n/a	
Being a medical professional	0.738; 2.09 (1.36, 3.22)	< 0.001^a^	0.770; 2.16 (1.34, 3.47)	0.001^a^	
Applied an elimination diet according to a doctor’s recommendations	0.147; 1.16 (0.72, 1.86)	0.545	n/a	n/a	
**Drinking large amounts of water increases milk production.**					FALSE
Parity	0.091; 1.1 (0.93, 1.29)	0.279	n/a	n/a	
Duration of breastfeeding the first child	0.043; 1.04 (1.03, 1.06)	< 0.001^a^	0.042; 1.04 (1.03, 1.06)	< 0.001^a^	
Applied an elimination diet during breastfeeding	−0.119; 0.82 (0.63, 1.07)	0.146	n/a	n/a	
Used elimination diet and replaced breastfeeding with infant formula	−0.743; 0.48 (0.25, 0.9)	0.022^a^	−0.563; 0.48 (0.25, 0.9)	0.091	
Being a medical professional	0.044; 1.04 (0.82, 1.34)	0.729	n/a	n/a	
Applied an elimination diet according to a doctor’s recommendations	−0.114; 0.89 (0.66, 1.21)	0.457	n/a	n/a	
**A breastfed child cries because of mother’s improper diet.**					FALSE
Parity	−0.266; 0.77 (0.5, 1.16)	0.211	n/a	n/a	
Duration of breastfeeding the first child	0.021; 1.02 (0.98, 1.06)	0.27	n/a	n/a	
Applied an elimination diet during breastfeeding	−1.589; 0.2 (0.11, 0.38)	< 0.001^a^	2.257; 0.1 (0.05, 0.22)	< 0.001^a^	
Used elimination diet and replaced breastfeeding with infant formula	−0.804; 0.45 (0.15, 1.3)	0.14	n/a	n/a	
Being a medical professional	−0.197; 0.82 (0.44, 1.53)	0.537	n/a	n/a	
Applied an elimination diet according to a doctor’s recommendations	0.626; 0.53 (0.27, 1.04)	0.066^a^	1.091; 2.98 (1.34, 6.62)	0.007^a^	
**Spicy food in mother’s diet causes colic in a breastfed infant.**					FALSE
Parity	0.032; 1.03 (0.79, 1.34)	0.81	n/a	n/a	
Duration of breastfeeding the first child	0.049; 1.05 (1.02, 1.08)	< 0.001^a^	0.047; 1.05 (1.02, 1.08)	< 0.001^a^	
Applied an elimination diet during breastfeeding	−1.327; 0.27 (0.18, 0.39)	< 0.001^a^	−2.112; 0.12 (0.07, 0.21)	< 0.001^a^	
Used elimination diet and replaced breastfeeding with infant formula	−0.271; 0.76 (0.34, 1.73)	0.516	n/a	n/a	
Being a medical professional	−0.086; 0.92 (0.62, 1.35)	0.665	n/a	n/a	
Applied an elimination diet according to a doctor’s recommendations	−0.555; 0.58 (0.38, 0.88)	0.011^a^	1.031; 2.8 (1.57, 5.0)	< 0.001^a^	
**Legumes, e.g. peas, in the mother’s diet cause bloating in the infant.**					FALSE
Parity	−0.173; 0.84 (0.65, 1.08)	0.18	n/a	n/a	
Duration of breastfeeding the first child	0.034; 1.03 (1.01, 1.06)	0.005^a^	0.030; 1.03 (1.01, 1.06)	0.017^a^	
Applied an elimination diet during breastfeeding	−1.383; 0.25 (0.17, 0.37)	< 0.001^a^	−2.392; 0.09 (0.05,0.15)	< 0.001^a^	
Used elimination diet and replaced breastfeeding with infant formula	−0.519; 0.59 (0.28, 1.25)	0.17	n/a	n/a	
Being a medical professional	−0.323; 0.72 (0.5, 1.05)	0.09	n/a	n/a	
Applied an elimination diet according to a doctor’s recommendations	− 0.465; 0.63 (0.41, 0.96)	0.031^a^	1.336; 3.8 (2.15, 6.72)	< 0.001^a^	
**Drinking coffee during lactation is allowed.**					TRUE
Parity	0.094; 1.1 (0.76, 1.6)	0.62	n/a	n/a	
Duration of breastfeeding the first child	0.013; 1.01 (0.98, 1.04)	0.414	n/a	n/a	
Applied an elimination diet during breastfeeding	−0.685; 0.5 (0.29, 0.86)	0.013^a^	−0.665; 0.51 (0.3, 0.88)	0.016^a^	
Used elimination diet and replaced breastfeeding with infant formula	1.039; 2.83 (0.38, 20.8)	0.308	n/a	n/a	
Being a medical professional	0.657; 1.93 (1.03, 3.62)	0.041^a^	0.635; 1.89 (1.0, 3.55)	0.049^a^	
Applied an elimination diet according to a doctor’s recommendations	−0.404; 0.67 (0.36, 1.22)	0.192	n/a	n/a	
**Children of mothers who drink coffee during lactation often wake up at night.**					FALSE
Parity	0.206; 1.23 (0.82, 1.85)	0.326	n/a	n/a	
Duration of breastfeeding the first child	0.005; 1.005 (0.97, 1.04)	0.783	n/a	n/a	
Applied an elimination diet during breastfeeding	−0.379; 0.68 (0.37, 1.25)	0.218	n/a	n/a	
Used elimination diet and replaced breastfeeding with infant formula	0.124; 1.13 (0.27, 4.79)	0.866	n/a	n/a	
Being a medical professional	−0.514; 0.6 (0.34, 1.06)	0.079	n/a	n/a	
Applied an elimination diet according to a doctor’s recommendations	0.555; 1.74 (0.73, 4.14)	0.21	n/a	n/a	
**Pimples and erythema on the baby’s face always indicate an allergy to milk proteins - the mother must eliminate dairy from her diet.**					FALSE
Parity	−0.163; 0.85 (0.7, 1.03)	0.1	n/a	n/a	
Duration of breastfeeding the first child	0.032; 1.03 (1.02, 1.05)	< 0.001^a^	0.035; 1.03 (1.02, 1.05)	< 0.001^a^	
Applied an elimination diet during breastfeeding	−0.906; 0.4 (0.3, 0.54)	< 0.001^a^	−0.895; 0.37 (0.23, 0.6)	< 0.001^a^	
Used elimination diet and replaced breastfeeding with infant formula	−0.916; 0.4 (0.23, 0.71)	0.002^a^	−0.385; 0.41 (0.23, 0.73)	0.225	
Being a medical professional	0.497; 1.64 (1.21, 2.24)	0.002^a^	0.564; 1.77 (1.25, 2.5)	0.001^a^	
Applied an elimination diet according to a doctor’s recommendations	−0.729; 0.48 (0.35, 0.67)	< 0.001^a^	0.212; 0.51 (0.36, 0.7)	0.438	
**During lactation, eating allergenic products, such as citrus, chocolate, nuts is forbidden.**					FALSE
Parity	−0.170; 0.84 (0.61, 1.16)	0.297	n/a	n/a	
Duration of breastfeeding the first child	0.047; 1.05 (1.01, 1.08)	0.004^a^	0.044; 1.05 (1.01, 1.08)	0.009^a^	
Applied an elimination diet during breastfeeding	−1.511; 0.22 (0.14, 0.36)	< 0.001^a^	−2.102; 0.12 (0.06, 0.23)	< 0.001^a^	
Used elimination diet and replaced breastfeeding with infant formula	−0.403; 0.67 (0.26, 1.73)	0.407	n/a	n/a	
Being a medical professional	−0.058; 0.94 (0.58, 1.53)	0.812	n/a	n/a	
Applied an elimination diet according to a doctor’s recommendations	−0.764; 0.47 (0.28, 0.77)	0.003^a^	0.810; 2.25 (1.16, 4.36)	0.016^a^	

*B* Coefficient, *OR* Odds Ratio, *CI* Confidence Interval, *n/a* non applicable

^a^statistically significant

### Dietary restriction during breastfeeding - practices and experience of respondents

During the course of lactation, 29.1% (*n* = 313) of all mothers followed an elimination diet, mostly due to medical recommendation (*n* = 223) and suspicion of allergy to cow’s milk proteins in infants (*n* = 212). In 16.9% of the above-mentioned mothers, dietary restrictions resulted in the cessation of breastfeeding. A statistically significant positive correlation was found between dietary restrictions during breastfeeding and the use of infant formula (*p* <  0.001). Among the medical staff, 15% admitted to having ever recommended exclusion of certain products from maternal diet as a preventive measure for future development of allergies in an infant. The statistical analysis revealed that parity (*p* <  0.001) and self-compliance with the elimination diet during lactation (*p* <  0.001) had a significant impact on recommending preventive dietary restrictions to mothers. There was no significant correlation between following (*p* = 0.237) nor recommending (*p* = 0.193) elimination diet and place of residence.

## Discussion

To the best of our knowledge, this is the first study which examines the beliefs of both medical staff and breastfeeding mothers in respect of rules of nutrition during lactation and on the effect of maternal diet in breast milk composition and infant’s health. The results of the study confirm that convictions regarding the negative effects of routine dietary habits during breastfeeding and the possibility of harmfulness of breast milk, affect maternal perception of nutrition and breastfeeding practices.

The main factor influencing respondents’ knowledge in respect of questioned statements was the duration of lactation. The findings of the other worldwide studies indicated that knowledge towards breastfeeding was strongly correlated with the duration of exclusive breastfeeding [15, 16]. In addition, breastfeeding practices were influenced by the mother’s knowledge of breastfeeding recommendations [16]. In the authors’ opinion, there is a two-way dependence between the knowledge of breastfeeding and the duration of lactation. The higher is the general knowledge (e.g. recommendations, properties of breast milk, short- and long-term effects of breastfeeding for both mothers and infants), the longer is the breastfeeding period, with emphasis on exclusive breastfeeding. Consequently, the longer the duration of lactation, the higher is maternal knowledge of particular aspects of breastfeeding – e.g. breastfeeding techniques, solutions for nursing difficulties, as well as maternal nutrition during lactation.

Moreover, the results of the survey indicate that the experience of following an elimination diet when breastfeeding determines mothers’ opinion regarding nutrition and its impact on breast milk quality as well as on the general condition of a breastfed child. It seems that there is a reciprocal relationship between dietary restrictions and the length of breastfeeding, and both of them might be influenced by other socioeconomic factors (e.g. breastfeeding support and acceptance, peer pressure, local customs or beliefs, attitudes towards breastfeeding, personal experience with breastfeeding a previous child, parity, comorbidities, level of education, providing facilities for breastfeeding in the mother’s place of employment – based on the survey results and the available worldwide literature [7–11, 15, 16]).

The proportions answering questions correctly were comparable between medical personnel and mothers practicing non-medical professions. This suggests that there may be a growing awareness and increase in nutritional and lactational knowledge among society, leading to a decreasing adherence to myths. Reduction of factors disturbing the process of lactation, especially peer pressure due to lactational misbeliefs, is likely to enhance the breastfeeding rates [7, 8, 11]. On the other hand, insufficient lactational knowledge among medical staff might lead to low-quality or lack of lactational counseling, thus might affect maternal knowledge of nutrition and lactation and further decisions related to infant feeding choices.

The relevance of adequate maternal nutrition and reasonable lactational support in promotion of breastfeeding has been emphasized worldwide [3, 5]. Furthermore, the crucial influence of hospital policy and medical staff assistance after delivery on breastfeeding rates has been reported. Moreover, the post-discharge surveillance by physicians, especially pediatricians should facilitate continuation of breastfeeding due to the current recommendation [5]. Psychosocial determinants, including breastfeeding attitudes, knowledge, and social support in breastfeeding were found to affect the initiation of breastfeeding and the duration of exclusive breastfeeding [15, 17].

Medical staff did not seem to have significantly better education than mothers in general. In addition, personal experience with breastfeeding had an influence on further counselling. Both groups of respondents were found with insufficient knowledge of relationships between maternal nutrients intake and resulting milk composition. Higher awareness of these relationships might have a positive effect on maternal dietary choices, adequate supplementation and high-quality nutritional counselling as a part of routine medical care. Also the practical knowledge of methods of how to enhance lactation and establish a good milk supply should be improved. On the other hand, high proportions of respondents who provided correct answers and the comparable prevalence of correct answers between both study groups suggest popularization of evidence based knowledge in Poland, and thus similar awareness of the validity of common myths and beliefs. Further training and education on particular issues of breastfeeding management is needed - not only among practicing medical staff, but in medical schools. Special concern should be given to increasing the knowledge of nutrition, physiology of lactation and differentia diagnosis of allergy to cow’s milk proteins.

Both mothers and medical professionals were found to have a moderately low level of knowledge of the impact of maternal nutrition on breast milk composition. Dietary habits do not seem to have a pronounced impact on particular components. In general, breast milk proteins and carbohydrates (mainly lactose) are the least affected by external variables and their concentrations depend primarily on the infant’s gestational age at birth and on the duration of lactation with regard to the infant’s postnatal age [3, 18]. According to the current knowledge, maternal dietary fat intake does not have any remarkable impact on total fat concentration in breast milk. However, it affects the breast milk fat quality, with significant alterations in the fatty acids profile [2, 3, 18]. The vitamin content of human milk is mainly dependent on maternal vitamin status and vitamin deficiency in the mother results in its low output to breast milk. What is more, the concentrations of vitamins are affected by dietary intake, and in a longer assessment, the higher supply, the higher concentration in breast milk [2, 18]. Similar correlations have been observed regarding selenium and iodine, but not e.g. iron, zinc, copper [2]. The results of the survey indicate that knowledge of these correlations should be improved. Nutritional counselling of breastfeeding mothers may be considered essential, and, as a further benefit, patient compliance may increase due to a better understanding of medical recommendations. This is especially significant in the medical care of vegetarians and vegans, who produce nutritionally adequate breast milk of similar composition to breast milk of mothers following a traditional diet, provided the obligatory supplementation of vitamin B12, omega-3 fatty acids and good adherence to dietary recommendations [19–21].

In general, there is no specific food to avoid when breastfeeding and no evidence in respect of a protective effect of a prophylactic maternal exclusion diet during pregnancy or during lactation on the occurrence of atopic diseases in infants [6]. General knowledge of this topic was reported by a high majority of respondents, which indicates growing awareness of current recommendations and diminishing prevalence of unscientific approaches to maternal diet while breastfeeding. When providing parental education and support, medical staff should also discuss the issue of the controversial impact of particular foods in maternal diet on breastfed infants’ intestinal disorders or fuss-cry behaviours. There is no apparent evidence regarding the association of cow’s milk, dairy products, chocolate, cruciferous vegetables and legumes with colic symptoms, and therefore mothers should not be advised to exclude these products prophylactically [22, 23]. In addition, parents should be informed that any type of facial rash in infants does not mean a straightforward diagnosis of allergy to cow’s milk proteins and the study revealed that breastfeeding mothers more often than medical staff attributed infantile skin lesions to allergy. Basically, the diagnosis of cow’s milk allergy might be considered if symptoms presented by a child cannot be explained with other conditions. Mild sensitization might be manifested with skin lesions, thus beside physical examination, a conscientious medical interview, including feeding history is essential for differential diagnosis [24, 25]. Allergy to cow’s milk proteins develops in 2–3% of infants in their first year of life [26]. However, based on the results of the study, mothers were most often recommended to start an elimination diet due to the suspicion of allergy to cow’s milk proteins in their infants, which suggest overdiagnosis of this ailment.

Based on the results of the study, drinking large amounts of water is believed to enhance lactation. In fact, there is no direct correlation between fluid intake and milk volume [8, 27]. As mothers might be worried about producing sufficient amounts of breastmilk for their infants, they need to receive adequate support and counselling, thus medical staff should be well-educated in the physiology of lactation and acquainted with indicators of successful breastfeeding.

### Limitations

As the study group included mainly internauts active on randomly selected discussing groups, the results should not be fully generalized to the Polish population. Another limitation is reliance on data provided by respondents. The unsupervised conditions of completing the questionnaire gave a possibility that respondents might have used additional information sources. On the other hand, lack of a supervisor eliminated interviewer-related errors. Further research on this topic in different regions of the world and among larger study groups, especially among medical staff is needed. We believe it might be helpful in increasing the quality of lactational counselling, as well as in identification of the main breastfeeding management issues and applying strategic solutions to increase the local breastfeeding rates.

## Conclusions

The conviction that preventive diet restrictions during lactation deliver beneficial effects is still present both among breastfeeding mothers and medical staff. Personal experience with dietary restrictions during the period of lactation affects further counselling to breastfeeding patients. In general, regardless of the practiced profession, the level of knowledge regarding maternal nutrition and its influence on lactation is quite good and comparable between breastfeeding mothers and medical staff. Growing awareness as well as nutritional and lactational knowledge within society might decrease adherence to myths. However, the awareness of the correlation between breast milk composition and dietary habits and the quality of diet should be improved. The quality of medical staff’s knowledge may affect the mothers’ knowledge in respect of nutrition and breastfeeding due to recommendations given while providing health care. Lack of reliable counselling, popularization of common lactational and nutritional misconceptions and insisting on dietary restriction without exact medical indications might be the factors limiting the duration of exclusive breastfeeding among Polish mothers, a topic that needs to be addressed by further research. There is the need to update the curricula and improve the competences of medical staff caring for pregnant and lactating women and their infants in order to promote medical staff as a reliable source of lactational knowledge.

## Data Availability

The datasets used and/or analyzed during the current study are available from the corresponding author on reasonable request.

## Abbreviations

AAP: American Academy of Pediatrics
DHA: Docosahexaenoic acid
DGE: German Nutrition Society
EPA: Eicosapentaenoic acid
ESPGHAN: European Society for Paediatric Gastroenterology Hepatology and Nutrition
FCN: Swiss Federal Commission for Nutrition
IOM: Institute of Medicine
IU: International unit
WHO: World Health Organization
